# Sociodemographic correlates of HIV drug resistance and access to drug resistance testing in British Columbia, Canada

**DOI:** 10.1371/journal.pone.0184848

**Published:** 2017-09-22

**Authors:** Genevieve Rocheleau, Conrado Franco-Villalobos, Natalia Oliveira, Zabrina L. Brumme, Melanie Rusch, Jeannie Shoveller, Chanson J. Brumme, P. Richard Harrigan

**Affiliations:** 1 Department of Medicine, University of British Columbia, Vancouver, Canada; 2 BC Centre for Excellence in HIV/AIDS, Vancouver, Canada; 3 Faculty of Health Sciences, Simon Fraser University, Burnaby, Canada; 4 Vancouver Island Health Authority, Victoria, Canada; 5 School of Population and Public Health, University of British Columbia, Vancouver, Canada; University of Cyprus, CYPRUS

## Abstract

Sociodemographic correlates of engagement in human immunodeficiency virus (HIV) care are well studied, however the association with accessing drug resistance testing (DRT) and the development of drug resistance have not been characterized. Between 1996–2014, 11 801 HIV patients accessing therapy in British Columbia were observed longitudinally. A subset of 9456 patients had testable viral load; of these 8398 were linked to census data. Sociodemographic (census tract-level) and clinical (individual-level) correlates of DRT were assessed using multivariable General Estimating Equation logistic regression adjusted odds ratios (aOR). The mean number of tests per patient was 2.1 (Q1-Q3; 0–3). Separately, any drug resistance was determined using IAS-USA (2013) list for 5703 initially treatment naïve patients without baseline resistance; 5175 were census-linked (mean of 1.5 protease-reverse transcriptase sequences/patient, Q1-Q3; 0–2). Correlates of detecting drug resistance in this subset were analyzed using Cox PH regression adjusted hazard ratios (aHR). Our results indicate baseline CD4 <200 cells/μL (aOR: 1.5, 1.3–1.6), nRTI-only baseline regimens (aOR: 1.4, 1.3–1.6), and unknown (therapy initiation before routine pVL in BC) baseline pVL (aOR: 1.8, 1.5–2.1) were among individual-level clinical covariates strongly associated with having accessed DRT; while imperfect adherence (aHR: 2.2, 1.9–2.5), low baseline CD4 count (aHR: 1.9, 1.6–2.3), and high baseline pVL (aHR: 2.0, 1.6–2.6) were associated with a higher likelihood of developing drug resistance. A higher median income (aOR: 0.83, 0.77–0.89) and higher percentage of those with aboriginal ancestry (aOR: 0.85, 0.76–0.95) were census tract-level sociodemographic covariates associated with decreased access to DRT. Similarly, aboriginal ancestry (aHR: 1.2, 1.1–1.5) was associated with development of drug resistance. In conclusion, clinical covariates continue to be the strongest correlates of development of drug resistance and access to DRT for individuals. Regions of high median income and high aboriginal ancestry were weak census-level sociodemographic indicators of reduced DRT uptake, however high aboriginal ancestry was the only sociodemographic indicator for development of drug resistance.

## Introduction

In resource rich settings with uninterrupted access to combination antiretroviral therapy (cART) and ongoing HIV-related care, human immunodeficiency virus (HIV) infection has become a manageable chronic illness with life expectancy approaching that of the general population [1]. However, the negative impact of drug resistance on treatment response is well-established [2–7] and the use of drug resistance testing to guide clinical decision-making has yielded improved treatment outcomes in randomized clinical trials [8–11]. As a result, drug resistance testing is the current standard of care in BC and elsewhere [12,13].

Despite these advances, research indicates low socioeconomic status not only increases vulnerability to HIV infection, but also impedes engagement and retention of HIV-infected persons in clinical care [14–22]. It is conceivable therefore that social and demographic factors associated with reduced access to HIV clinical services such as drug resistance testing could lead to elevated risks of drug resistance, and thus adverse health outcomes, in certain demographic groups. However, studies explicitly linking HIV drug resistance, and access to HIV drug resistance testing to sociodemographic factors are lacking. This research could therefore inform a more nuanced understanding of the changing HIV epidemic. The objective of this study is to examine the sociodemographic correlates of the development of drug resistance and access to drug resistance testing in a province-wide sample of HIV-positive patients receiving cART.

## Methods

### Accessing drug resistance testing cohort

#### Data collection

In the province of British Columbia (BC), Canada, antiretroviral (ARV) therapy is distributed through the provincial Drug Treatment Program, operated through the BC Centre for Excellence in HIV/AIDS [11]. This study followed 11 801 Drug Treatment Program patients between 1996–2014. Antiretroviral medication was prescribed according to BC guidelines and was provided free of charge to the patient. Administrative data such as prescriptions and lab test results were collected on an ongoing basis until the patient was lost to follow-up by moving out of BC, passing away, or entering a clinical trial. In these cases, patients were censored at the most recent data collected. Human blood specimen, data collection and use has approval under the University of British Columbia Research Ethics Board at Providence Health Care Research Institute. Due to the administrative nature of the data, Research Ethic Board waived the requirement for consent under protocol H05-50123. This study was reported in accordance with the STROBE statement [23].

#### Drug resistance testing

The BC treatment guidelines recommend testing for ARV resistance in all individuals prior to therapy as well as that at virologic failure (pVL>250 copies/mL) [11]. In each calendar year patients were considered eligible for testing when ≥1 sample was above the lower limit of detection of the DRT assay in use at that time (usually pVL >250 copies/mL). Patients with a physician-ordered DRT result available were considered to have accessed testing during that calendar year. Among the 11 801 patients initially considered, 9456 had eligible pVL in any year of the study. Among those eligible, a mean number of 2.1 (Q1-Q3; 0–3.0) resistance tests were ordered per patient over the course of follow-up.

#### Linking of census data and clinical data

Patient postal code or city of residence at time of Drug Treatment Program enrollment determined their census tract. Clinical data was linked with census tract-level data from the Census Canada Survey that was conducted closest to Drug Treatment Program enrollment date (census 1996, 2001, 2006, or 2011). If insufficient information was available to determine census tract, census metropolitan area/census agglomeration (or local health area) was used. Among patients with eligible pVL, 8398 had clinical data linked to census tract data. See S3 Table for a comparison of characteristics of included and excluded individuals.

#### Plasma viral load testing

Testing is free of charge in BC when ordered by a physician, and occurs regularly as part of routine patient care [11]. Testing was completed at the St. Paul’s Hospital Virology Laboratory using Roche Molecular Diagnostics kits.

#### Quantification of adherence to prescribed drug regimen

Prescription refill percentage, obtained from Drug Treatment Program ARV prescription records and calculated as the number of days with antiretroviral drugs dispensed divided by the number of days of follow up in the first year on therapy, was used as a crude estimate of adherence and has been demonstrated as a good predictor of future adherence [24].

#### Statistical methods

Unadjusted odds ratios (uOR), adjusted odds ratios (aOR), and 95% confidence intervals (CIs) were determined by Generalized Estimating Equations (GEE) logistic regression. The optimal multivariable explanatory model was selected using an Akaike Information Criterion (AIC)-based backward elimination procedure.

Covariate selection was completed through backward elimination to minimize the Quasi-likelihood Information Criterion (QICu).

The model was adjusted for individual-level biological, transmission risk group and clinical covariates. All covariates were treated as categorical, and included sex at birth (male, female), transmission risk factor (men who have sex with men (MSM), people who inject drugs (PWID), heterosexual), ever diagnosed with hepatitis C (HCV), drug in first recorded regimen (Only nRTI -including mono-, dual-, or triple-therapy; NNRTI as third drug in regimen; or PI as third drug in regimen), suboptimal adherence (<95% for first year on therapy), age at enrollment (years), baseline CD4 count (cells/μL), baseline pVL (copies/mL)–including baseline pVL unknown (first ARV before 1997; prior to baseline pVL testing implementation in BC), whether a patient was eligible for drug resistance test (per year), and physician experience (number of HIV patients treated in the previous two years). Models were adjusted for unknown baseline CD4, unknown baseline pVL and unknown adherence, but these are not shown in the final figure for readability and due to low N in these categories. See S7 Table for the comprehensive model.

Sociodemographic covariates were calculated at the census tract-level, including percentage of one-family households among total number of private households in the area (per 10% increment), percentage of single people (per 10%), population density (per 10k inhabitants), percentage of immigrants (per 10%), median income (per $10k), percentage with post-secondary certificate (per 10%), unemployment rate (per 10%), and percentage of census tract residents that have aboriginal ancestry (<5%, 5%-<10%, and ≥10%).

Pearson correlation coefficients (PCC) were calculated for all possible pairs of census-level covariates. In cases where strong correlation was observed between two covariates, median income, percent of single family households, percent of immigrants, and the percent of census tract residents with aboriginal ancestry were given priority; thus avoiding multicollinearity. Data manipulation was done in SAS 9.4 (SAS Institute, Cary NC) and statistical analyses in R 3.3.1 [25,26].

### Development of drug resistance cohort

#### Data collection

The HAART (Highly Active Antiretroviral Therapy) Observational Medical Evaluation and Research (HOMER) cohort is a subset of the Drug Treatment Program (N = 11,801). HOMER participants are ≥19 year old, initially treatment naïve individuals who, did not harbor baseline resistance, started HAART (defined as triple-drug combination therapy with regimens consisting of two nucleos(t)ide reverse transcriptase inhibitors (NRTIs) plus a protease inhibitor (PI), non-nucleoside reverse transcriptase inhibitor (NNRTI), or integrase inhibitor (INI)), between 1996–2013, had baseline pVL and baseline CD4 cell count measured within six months prior to therapy initiation, and had a minimum of one year of follow-up (N = 5,703).

#### Clinical data and census linkages

Linkage was conducted in the same manner as the access to DRT cohort described above. Within HOMER, 5175 patients had clinical data linked to census tract data. See S6 Table for a comparison of included and excluded individuals. Plasma viral load testing and adherence estimation were conducted in the same manner as the access to DRT cohort described above.

#### Drug resistance testing

Resistance testing in BC is provided at no cost to the patient. Drug resistance genotyping was attempted on all available plasma samples; these included physician-ordered tests and genotyping performed for research purposes. HIV RNA was extracted and genotyping of the protease and reverse transcriptase genes was performed as previously described (mean 1.5; Q1-Q3 0–2.0 resistance tests/patient) [27–29]. Resistance was defined as the presence of ‘key’ resistance mutation from the IAS-USA (2013) mutation list [30]. Detection of any resistance was considered the endpoint and patients were censored after the date of the positive resistance test. Patients without baseline resistance tests were assumed not to harbor resistance. Integrase resistance was not included since no individuals in this study initiated therapy with integrase inhibitors.

#### Statistical methods

Unadjusted hazard ratios (uHR), adjusted hazard ratios (aHR), and 95% confidence intervals (CI) of time to any drug resistance among those with eligible pVLs were determined by Cox proportional hazards (PH) regression. Covariate selection was completed through backward elimination to minimize the Quasi-likelihood Information Criterion (QICu). Violation of the proportional hazard assumption was tested using the global test for proportionality.

Covariates that were accounted for in the development of drug resistance model were identical to the access to DRT model, with the exception of the following individual-level covariates: eligibility for drug resistance test (per year) was replaced with year of therapy initiation, drug in first recorded regimen (Only nRTI including mono-, dual-, or triple-therapy; NNRTI as third drug in regimen; or PI as third drug in regimen) was replaced with third drug in baseline regimen (NNRTI, or PI), and within the baseline pVL category; baseline pVL unknown (first ARV before 1997) was removed as baseline viral load was a HOMER inclusion criterion. The comprehensive model can be found in the S8 Table. The procedure described in the access to DRT cohort statistical methods section was followed for covariate multicollinearity.

## Results

### Accessing drug resistance testing cohort

#### Cohort description

Between 1996–2014, 11 801 HIV-positive patients were observed longitudinally; 9456 had ≥1 pVL test above the limit of detection for DRT in that era (usually pVL >250 copies/mL) after therapy initiation and were therefore eligible for a physician-ordered DRT. Due to missing data, census data could not be linked to clinical data for 1058 participants, resulting in a final tally of 8398 individuals included in this analysis. Baseline characteristics of those included were found to be statistically different from those excluded, with the exception of sex at birth (P = 0.078); see S3 Table. Most patients were male at birth (82%), and over one third were MSM (34%), PWID (35%), or co-infected with HCV (37%). The majority was ≥95% adherent to the prescribed drug regimen during the first year of therapy (54%), and half had ever received a physician-ordered DRT (49%). The median age at study entry was 40 years (1^st^ quartile-3^rd^ quartile; 33–47). See Table 1 for complete description of clinical characteristics. See Table 2 for census-tract level sociodemographic characteristics. Among participants eligible for a drug resistance test, the proportion of those accessing testing increased gradually over the course of the study (S1 Fig), from 29% in 1996 to 54% in 2013.

**Table 1:** Cohort clinical baseline characteristics.

Baseline Clinical Characteristics	Accessing DRT	Any Drug Resistance
N (%)	8398 (100)	5175 (100)
Sex at birth		
Male—n (%)	6916 (82)	4232 (82)
Female—n (%)	1482 (18)	943 (18)
MSM risk		
No—n (%)	3501 (42)	2303 (45)
Yes—n (%)	2842 (34)	1491 (29)
Unknown—n (%)	2055 (25)	1381 (27)
Heterosexual risk		
No—n (%)	4108 (49)	2380 (46)
Yes—n (%)	1773 (21)	1137 (22)
Unknown—n (%)	2517 (30)	1658 (32)
PWID risk		
No—n (%)	3852 (46)	2325 (45)
Yes—n (%)	2919 (35)	1888 (36)
Unknown—n (%)	1627 (19)	962 (19)
Hepatitis C positive		
No—n (%)	4266 (51)	2734 (53)
Yes—n (%)	3132 (37)	2109 (41)
Unknown—n (%)	1000 (12)	332 (6)
Baseline CD4		
<200 cells/μL—n (%)	3273 (39)	2310 (45)
200-<350 cells/μL—n (%)	2516 (30)	1558 (30)
≥350 cells/μL—n (%)	2505 (30)	1307 (25)
Baseline Viral Load		
<10,000 copies/mL—n (%)	958 (11)	553 (11)
10,000-<100,000 copies/mL—n (%)	2865 (34)	2118 (41)
≥100,000 copies/mL—n (%)	3043 (36)	2504 (48)
Baseline regimen third drug class		
PI—n (%)	3679 (44)	3064 (59)
NNRTI—n (%)	2472 (29)	2111 (41)
nRTI Only–n (%)	2077 (25)	N/A
Other–n (%)	170 (2)	N/A
Adherence in first 12 months of therapy <95%		
No—n (%)	4543 (54)	3215 (62)
Yes—n (%)	3550 (42)	1960 (38)
Patients ever having a drug resistance test		
No—n (%)	4271 (51)	2904 (56)
Yes—n (%)	4127 (49)	2271 (44)
Median year of ARV initiation (Q_1_-Q_3_)	2002 (1997–2008)	2005 (2000–2009)
Median age at ARV initiation in years (Q_1_-Q_3_)	40 (33–47)	34 (31–48)

**Table 2:** Sociodemographic census tract-level characteristics of access to drug resistance testing cohort and development of drug resistance cohort.

Sociodemographic Census Tract-Level Characteristics	Accessing DRT—Median (Q1-Q3)	Any Drug Resistance—Median (Q1-Q3)
Percentage of single-family households	51 (30–66)	53 (30–66)
Population density (per 10K)	5150 (2560–10 800)	5150 (2500–10 500)
Percentage of immigrants	31 (21–39)	31 (21–40)
Median income ($)	23 200 (19 200–27 300)	24 300 (20 000–28 000)
Percentage of single people	38 (30–54)	37 (30–54)
Percentage with post secondary certification	53 (44–64)	54 (44–65)
Percentage unemployed	63 (56–67)	63 (56–68)
Percentage aboriginal ancestry	3.0 (1.0–5.0)	3.0 (1.0–6.0)

#### Odds ratios of correlates for accessing drug resistance testing

**Fig 1:**
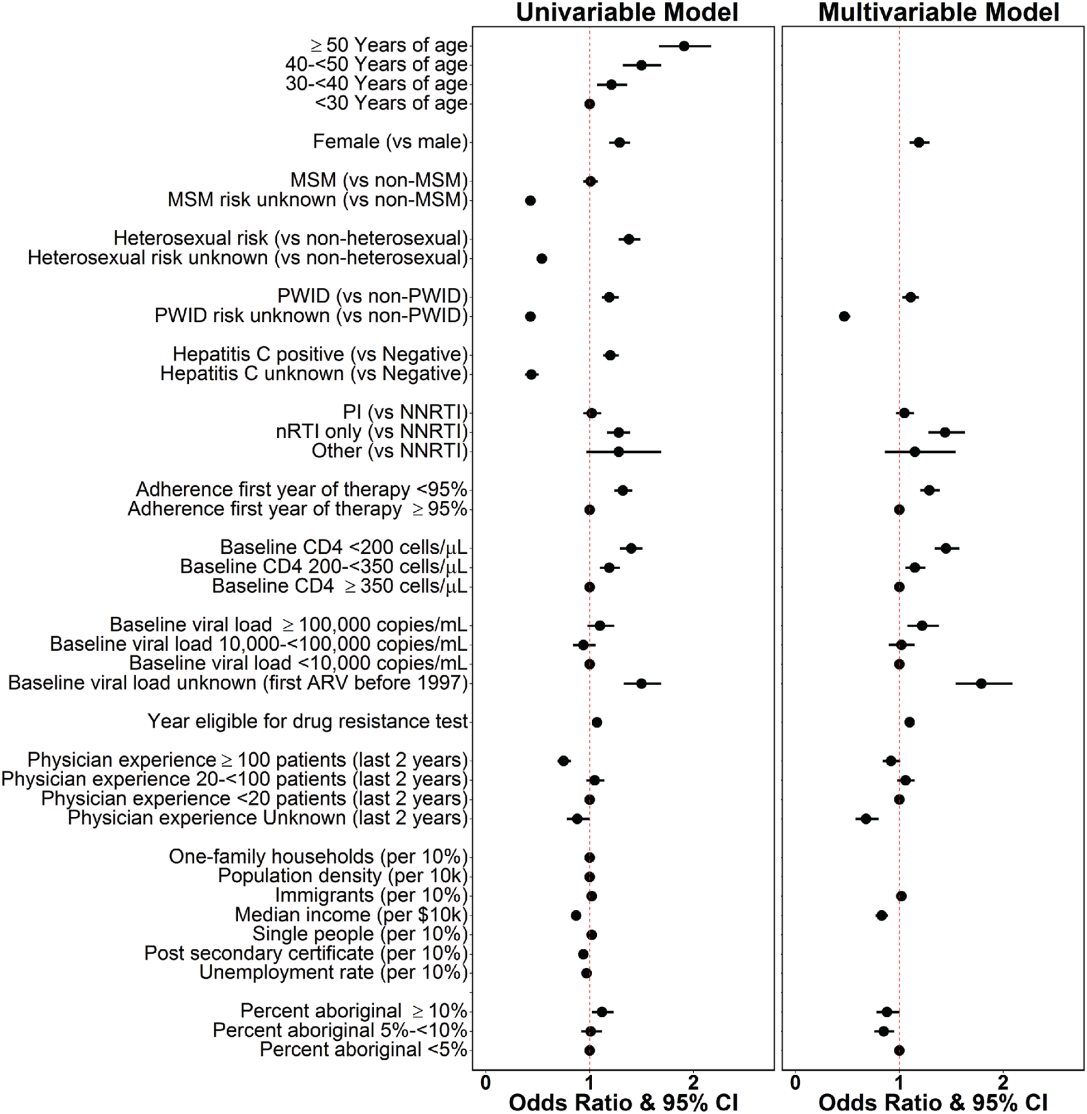
Odds ratio and 95% confidence intervals assessing the odds of accessing drug resistance testing, by model. Odds Ratios do not exist for covariates not selected under the multivariable model (see Methods).

The univariable and multivariable ORs for the covariates (see Methods) included in GEE logistic regression are presented in Fig 1. Among the sociodemographic covariates as determined from census tract-level data, every $10k increase in median income was associated with 17% lower odds ratio for accessing drug resistance testing (aOR: 0.83, 0.77–0.89), while census tracts with 5-<10% of people reporting aboriginal ancestry were associated with 15% lower odds ratio for accessing drug resistance testing when compared regions with <5% of people self-reporting aboriginal ancestry (aOR: 0.85, 0.76–0.95).

Among the individual-level clinical covariates, odds ratios associated with access to drug resistance testing were 20% higher for women compared to men (aOR: 1.2, 1.1–1.3), 53% lower for those with unknown PWID status (aOR: 0.47, 0.42–0.53), 50% higher when baseline CD4 was <200 cells/μL (aOR: 1.5, 1.3–1.6), 20% higher for those with baseline CD4 of 200-<350 cells/μL (aOR: 1.2, 1.1–1.3), 80% higher when baseline pVL was unknown due to ARV initiation before pVL testing becoming available in 1997 (aOR: 1.8, 1.5–2.1), 40% higher among those with first recorded regimen as mono-, dual-, or triple-nRTIs (aOR: 1.4, 1.3–1.6), and 30% higher for those with adherence <95% (aOR: 1.3, 1.2–1.4). Baseline pVL >100,000 copies/mL (aOR: 1.2, 1.1–1.4) and physicians with unknown amount of experience treating HIV (aOR: 0.68, 0.58–0.80) were significant only after adjusting for other covariates.

Low adherence has been associated with low socio-economic status as well as PWID and the development of drug resistance, thus accounting for adherence could theoretically mask effects of these covariates [31,32]. However, we found that accounting for <95% adherence did not substantially change the results of the model, therefore adherence was included (see S1 Table).

### Development of drug resistance cohort

#### Cohort description

Data on 5175 eligible individuals (see Methods) between 1996–2014 was analyzed retrospectively. Baseline characteristics of those excluded were found to be statistically different from those included (see S6 Table). Similar to the access to DRT cohort described above, 82% of patients were male at birth, and 36% were PWID. Only 29% were MSM, while 41% were co-infected with HCV. Overall 62% were ≥95% adherent, but only 44% had ever been ordered a drug resistance test by a physician. The median age was 41 years (1^st^ quartile-3^rd^ quartile; 34–48). See Table 1 for complete description of clinical characteristics. See Table 2 for complete description of census-tract level sociodemographic characteristics.

#### Hazard ratios of correlates for development of drug resistance

**Fig 2:**
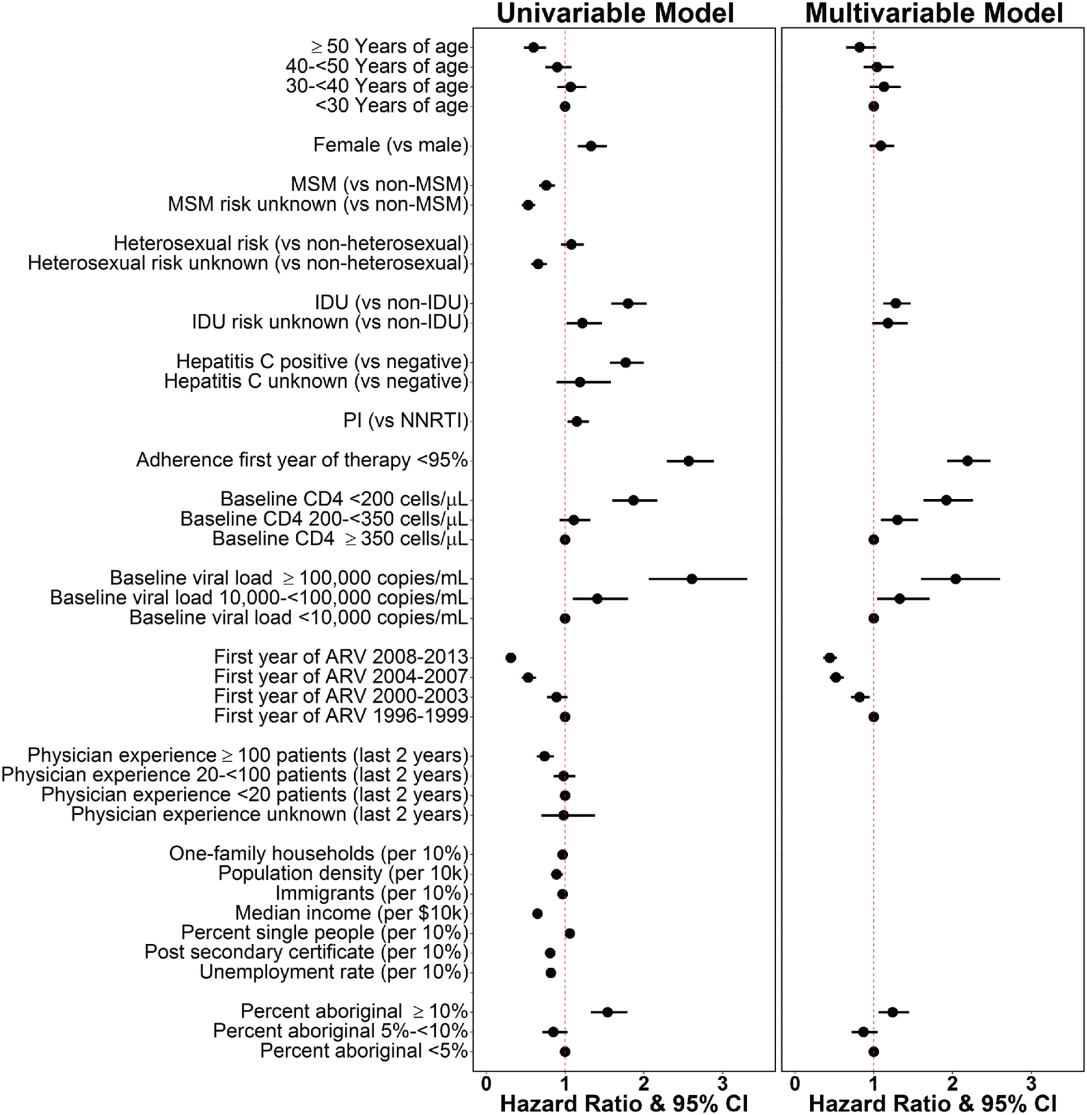
Hazard ratio and 95% confidence intervals assessing the likelihood of developing drug resistance in a treatment naïve cohort subset, by model. Hazard Ratios do not exist for covariates not selected under the multivariable model (see Methods).

The univariable and multivariable HRs for the covariates in the Cox PH regression are presented in Fig 2. The global test for proportionality gave a non-significant p-value (P = 0.109), also the interaction between time and each individual variable gave non-significant p-values ranging from 0.072 to 0.838, showing no evidence the proportional hazard assumption was violated. A higher proportion of residents reporting aboriginal ancestry was the only census tract-level sociodemographic covariate significantly associated with the development of drug resistance. Individuals in a census tract with ≥10% people self-reporting aboriginal ancestry were 20% more likely to develop drug resistance compared to regions with <5% self-reported aboriginal ancestry (aHR: 1.2, 1.1–1.5).

Among individual-level clinical covariates, the likelihood of developing drug resistance was 90% higher in individuals with baseline CD4 <200 cells/μL (aHR: 1.9, 1.6–2.3) and 30% higher for those were 200-<350 cells/μL (aHR: 1.3, 1.1–1.6) compared to those with ≥350 cells/μL, 100% higher among those with baseline pVL >100,000 copies/mL compared to those with <10,000 copies/mL (aHR: 2.0, 1.6–2.6), 30% higher in PWIDs (aHR: 1.3, 1.1–1.5), and 120% higher among those with <95% adherence (aHR: 2.2, 1.9–2.5). More recent ARV initiation was associated with decreased likelihood of developing drug resistance. Individuals who initiated therapy between 2000–2003 were 18% less likely to develop drug resistance compared to those who started between 1996–1999 (aHR: 0.82, 0.71–0.95); whereas, participants who initiated therapy between 2004–2007 (aHR: 0.52, 0.44–0.62), and 2008–2013 (aHR: 0.44, 0.36–0.53) were 48% and 56% less likely to develop drug resistance, respectively. Baseline CD4 200-<350 cells/μL, and those who first started ARVs between 2000–2003 became significant only after adjusting for other covariates. These results were broadly consistent when broken down by categories of drug resistance; emtricitabine/lamivudine (3TC/FTC) (S2 Fig), non-nucleoside reverse transcriptase inhibitors (NNRTI) (S3 Fig), other nucleoside reverse transcriptase inhibitors (nRTI) (S4 Fig), and protease inhibitors (PI) (S5 Fig).

Similar to the access to DRT model, adherence was tested as a confounder. Accounting for those who had <95% adherence did not substantially change the results (see S2 Table), therefore adherence was included in the final model.

## Discussion

This longitudinal observational study describes an exploratory analysis of sociodemographic correlates of drug resistance and access to drug resistance testing among patients receiving ARVs in BC between 1996–2014. The sociodemographic variables were determined using census tract-level data, while biological, transmission risk group and clinical variables used individual-level data. The results indicate that living in census tracts with high median income and high rates of aboriginal ancestry remain weakly correlated with decreased access to drug resistance testing after adjusting for clinical factors. However, only census-tracts reporting higher proportion of aboriginal ancestry had an elevated likelihood of developing HIV drug resistance. The combined census-level sociodemographic results indicate census-tracts with a high proportion of aboriginal ancestry may benefit from targeted public health interventions, such as more DRT-specific physician training or public awareness campaigns regarding HIV drug resistance.

The difference in effect size between sociodemographic and clinical covariates associated with access to drug resistance testing was notable; odds ratios of clinical covariates had up to 4 times stronger effect size than sociodemographic covariates. A similar disparity was observed in development of drug resistance; clinical covariates had between 4.5–6 times larger effect size than the strongest sociodemographic covariate. The results suggest that at the individual-level, clinical correlates are better determinants for the development of drug resistance or access to drug resistance testing in BC compared to census-level sociodemographic covariates.

More recent ARV initiation was associated with decreased likelihood of developing drug resistance. This is likely due to multiple factors, potentially including patient management, cohort-wide adherence, and improvements in regimens resulting in greater genetic barriers to resistance. Research expanding upon this finding has recently been published, but more work is required to determine causation [33].

PWIDs did not access resistance testing significantly more than non-PWIDs in this study. This result is counterintuitive and could result from effect modification, as imperfect adherence, development of drug resistance, and low median income have been previously associated with PWID [33–35]. Imperfect adherence has been strongly associated with the development of acquired drug resistance, leading to elevated viral load; the BC primary guidelines for the treatment of HIV recommends a drug resistance test be ordered under these circumstances [13,33,36]. Therefore adherence and drug resistance are on the causal pathway for drug resistance testing. To test for effect modification, the models were stratified by PWID status: No strong differences were found between PWID and non-PWID (see S4 and S5 Tables).

Aboriginal ancestry was associated with moderately lower access to testing as well as higher likelihood of developing drug resistance [37]. These results highlight the complex ways indigenous peoples interact with HIV-related health care, and more work is needed to address barriers to HIV care and related co-morbidities indigenous peoples face in Canada. Living in a census-tract with high median income was also marginally associated with reduced access to DRT. This finding does not support our hypothesis that low socioeconomic standing is associated with barriers to care. This is counter to results that have been reported in other areas of HIV research [19–22,38].

Nosyk et al (2013) observed access to HIV related care in BC has improved between 1996–2011; infected, undiagnosed HIV cases dropped 36–58%, while viral suppression increased from 1% to 35%, and individuals who are fully adherent but not virally suppressed dropped from 95% to 22% [39]. Our research implies these improvements in care access and retention occurred relatively equally over different sociodemographic strata. Potential causes of these results requires further, more detailed study. While no studies to our knowledge have directly investigated sociodemographic correlates of HIV drug resistance, previous studies have reported no strong association between MDR-TB and sociodemographic covariates in both high income and low income settings [40–44].

A limitation of this study was missing census data–as many as 373 patients had census data linked, but had incomplete or absent data for a given census question resulting from the change to a voluntary National Household Survey in 2011. Due to the low quality of data collected from a voluntary survey some data was deemed not fit for release, or flagged to be used with caution [45]. Additionally, those excluded were statistically different from those included in both cohorts of this study, which may bias the results. Drug resistance monitoring was not included in the clinical guidelines until the early 2000s, potentially skewing the results [46,47]. The extrapolation of results are limited to settings where testing is free of charge. Since sociodemographic data was gathered at the census tract level, there exists potential for ecological fallacy; this should be borne in mind when interpreting the results. Especially in areas of vast socioeconomic disparities, such as the downtown eastside of Vancouver, census-level variables are less likely to accurately describe the population, thus confounding the results. Ecological fallacy does not apply for clinical variables, as this data was gathered for each individual patient.

Our results suggest regions with high proportion of people with aboriginal ancestry may experience increased barriers to accessing testing and be at higher risk of developing resistance. Working with indigenous communities to promote culturally sensitive and appropriate programs could help to reduce some of these barriers. However, vast disparities in drug resistance were not observed between sociodemographic strata. The complex relationships between sociodemographic covariates make statistical modeling and interpretation a challenge. While evidence exists linking infectious diseases with poor socio-economic standing, there are few examples for drug resistant variants of HIV. Clinical covariates, particularly low CD4 cell count, high pVL, and imperfect adherence continue to be effective individual-level predictors of developing drug resistance and access to drug resistance testing across all sociodemographic strata.

## Supporting information

S1 Fig Percentage of patients accessing drug resistance testing among eligible participants, per year in BC. Patients were considered eligible when plasma viral load (pVL) was above the lower limit of detection of the drug resistance test in a calendar year. This changed year to year, but was generally higher than pVL of 250 copies/mL. Patients were considered to have accessed testing when a physician ordered a drug resistance test.
(TIF)

S2 Fig Hazard ratio and 95% confidence intervals assessing the likelihood of developing 3TC/FTC resistance in a treatment naïve cohort subset, by model. (TIF)

S3 Fig Hazard ratio and 95% confidence intervals assessing the likelihood of developing NNRTI resistance in a treatment naïve cohort subset, by model. (TIF)

S4 Fig Hazard ratio and 95% confidence intervals assessing the likelihood of developing “other” nRTI resistance (excluding 3TC/FTC) in a treatment naïve cohort subset, by model. (TIF)

S5 Fig Hazard ratio and 95% confidence intervals assessing the likelihood of developing PI resistance in a treatment naïve cohort subset, by model. (TIF)

S1 Table Adjusted odds ratios of GEE logistic regression models of access to drug resistance testing with adherence included in the model, and without adherence. (DOCX)

S2 Table Adjusted hazard ratios of Cox PH logistic regression models of development of drug resistance with adherence included in the model, and without adherence. (DOCX)

S3 Table Access to drug resistance testing (DRT) cohort; comparison between excluded and included individuals. Individuals were excluded due to missing data resulting in the inability to link census data to clinical data, therefore we were not able to compare differences between census-level sociodemographic data. Cohorts were compared using Chi-squared test.
(DOCX)

S4 Table Adjusted Odds Ratios of GEE logistic regression models for access to drug resistance testing, stratified by people who inject drugs (PWID). Individuals with unknown PWID status were excluded from this analysis (N = 1627).
(DOCX)

S5 Table Adjusted Hazard Ratios of Cox PH logistic regression models for the development of drug resistance stratified by people who inject drugs (PWID). Individuals with unknown PWID status were excluded from this analysis (N = 962).
(DOCX)

S6 Table Development of drug resistance cohort; comparison between excluded and included individuals. Individuals were excluded due to missing data resulting in the inability to link census-level sociodemographic data to individual clinical data, therefore we were not able to compare differences between census-level sociodemographic data. Cohorts were compared using Chi-squared test.
(DOCX)

S7 Table Access to drug resistance testing cohort; comprehensive univariable and multivariable models using GEE logistic regression. (DOCX)

S8 Table Development of drug resistance cohort; comprehensive univariable and multivariable models using Cox proportional hazards regression. (DOCX)
