# Supplementary figures and images for: Sociodemographic correlates of HIV drug resistance and access to drug resistance testing in British Columbia, Canada
Source: PLoS One. 2017 Sep 22;12(9):e0184848. doi: 10.1371/journal.pone.0184848 (PMC5609746; doi:10.1371/journal.pone.0184848)

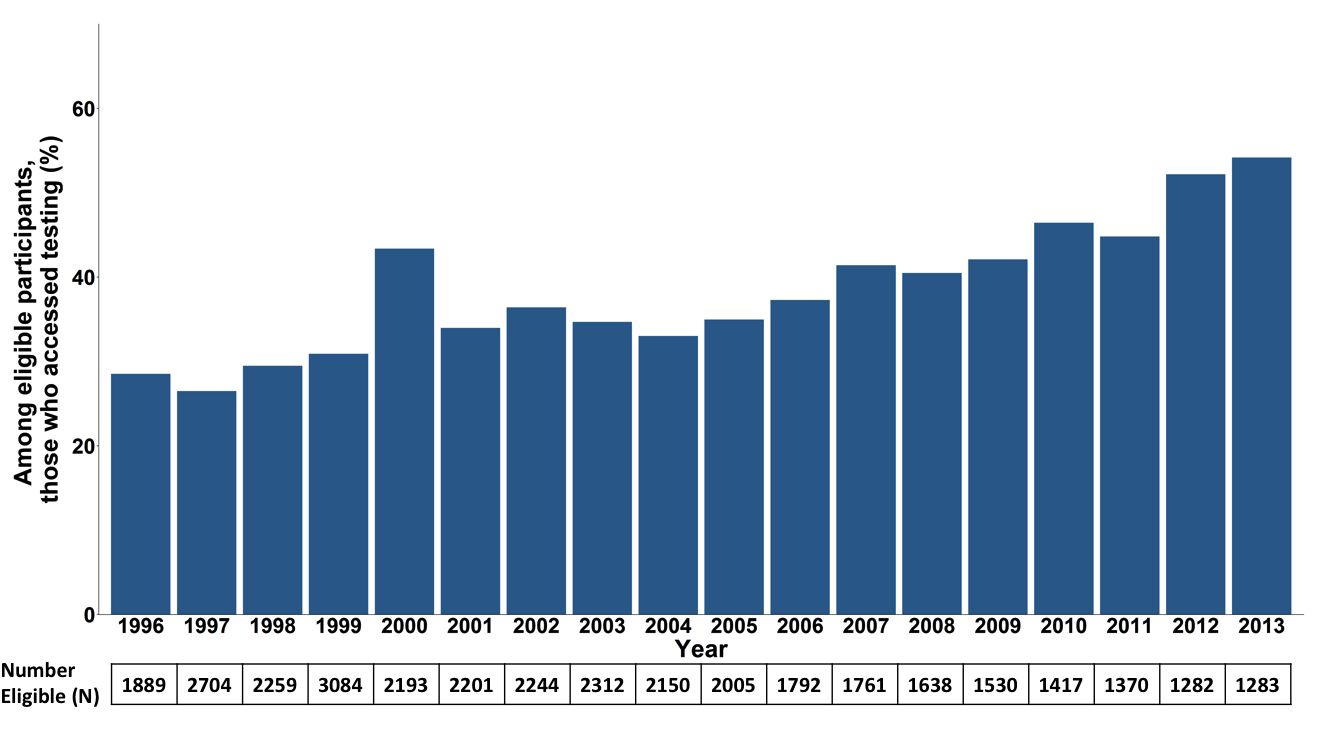

Supplement: S1 Fig — Patients were considered eligible when plasma viral load (pVL) was above the lower limit of detection of the drug resistance test in a calendar year. This changed year to year, but was generally higher than pVL of 250 copies/mL. Patients were considered to have accessed testing when a physician ordered a drug resistance test. (TIF) [file pone.0184848.s001.tif]

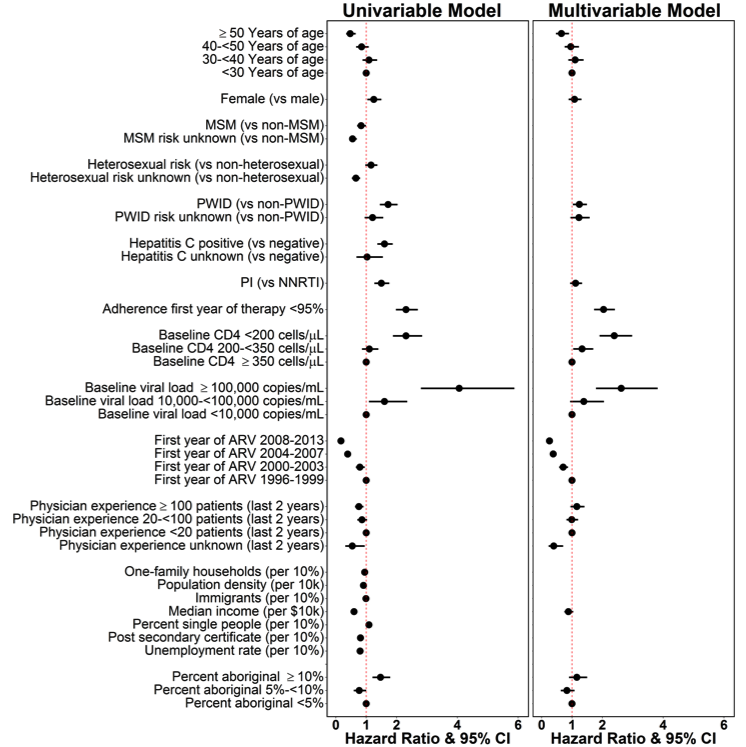

Supplement: S2 Fig — (TIF) [file pone.0184848.s002.tif]

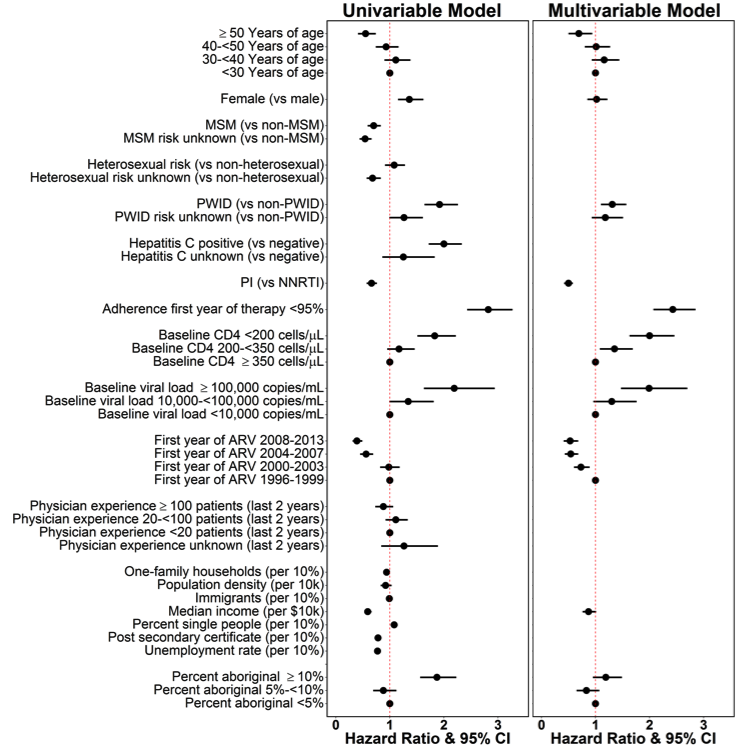

Supplement: S3 Fig — (TIF) [file pone.0184848.s003.tif]

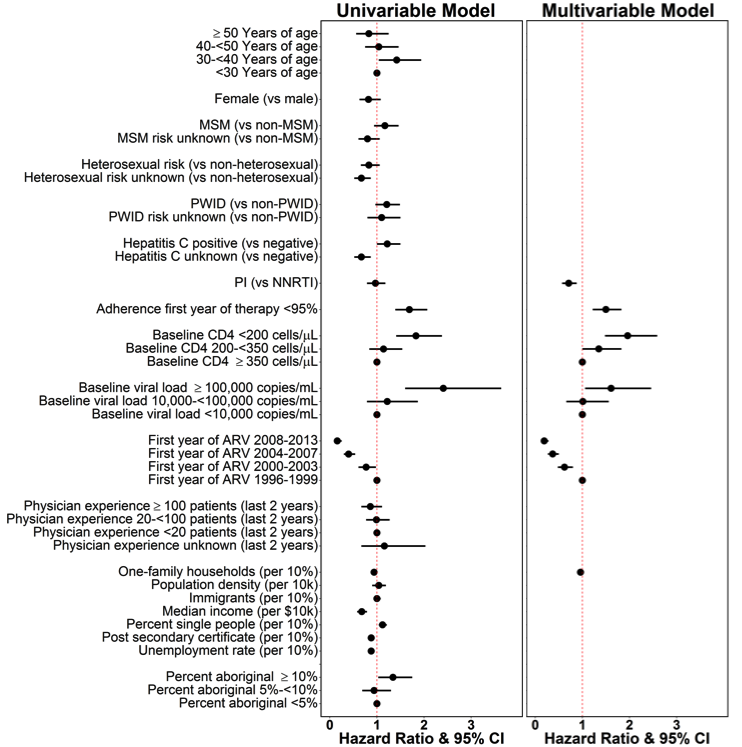

Supplement: S4 Fig — (TIF) [file pone.0184848.s004.tif]

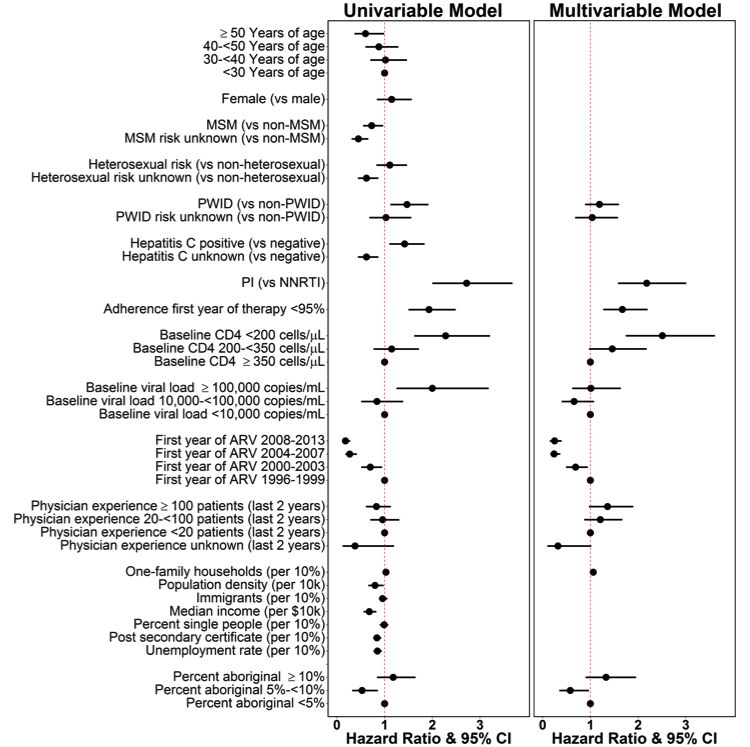

Supplement: S5 Fig — (TIF) [file pone.0184848.s005.tif]
