# Supplementary material for: Sociodemographic correlates of HIV drug resistance and access to drug resistance testing in British Columbia, Canada
Source: PLoS One. 2017 Sep 22;12(9):e0184848. doi: 10.1371/journal.pone.0184848 (PMC5609746; doi:10.1371/journal.pone.0184848)
Supplement: S1 Table — (DOCX) [file pone.0184848.s006.docx]

| **Multivariable Covariates of Accessing Drug Resistance Testing** | **Without Adherence – aOR (95% CI) N=8398** | **With Adherence – aOR (95% CI) N=8398** |
| --- | --- | --- |
| Sex |  |  |
| Female (vs Male) | 1.2 (1.1-1.3) | 1.2 (1.1-1.3) |
| Hepatitis C |  |  |
| Positive (vs Negative) | 1.2 (1.1-1.4) | 0.92 (0.80-1.1) |
| Unknown (vs Negative) | 0.38 (0.30-0.47) | 0.35 (0.25-0.49) |
| Baseline regimen third drug class |  |  |
| PI (vs NNRTI) | 1.1 (0.96-1.1) | 1.1 (0.97-1.1) |
| nRTI Only (vs NNRTI) | 1.5 (1.3-1.6) | 1.4 (1.3-1.6) |
| Other (vs NNRTI) | 1.2 (0.90-1.6) | 1.2 (0.86-1.5) |
| Adherence in first 12 months of therapy <95% (vs ≥95%) | Not Selected | 1.3 (1.2-1.4) |
| Baseline CD4 |  |  |
| <200 cells/μL | 1.4 (1.3-1.6) | 1.5 (1.3-1.6) |
| 200-<350 cells/μL | 1.1 (1.1-1.2) | 1.2 (1.1-1.3) |
| ≥350 cells/μL | Reference | Reference |
| Baseline pVL |  |  |
| ≥100,000 copies/mL | 1.1 (1.0-1.3) | 1.2 (1.1-1.4) |
| 10,000-<100,000 copies/mL | 0.96 (0.85-1.1) | 1.0 (0.90-1.2) |
| <10,000 copies/mL | Reference | Reference |
| Eligible for drug resistance test (per year) | 1.1 (1.1-1.1) | 1.1 (1.1-1.1) |
| Physician experience (last 2 years) |  |  |
| ≥100 patients | 0.90 (0.82-0.99) | 0.92 (0.84-1.0) |
| 20-100 patients | 1.1 (0.98-1.2) | 1.1 (0.98-1.2) |
| Unknown | 0.99 (0.87-1.1) | 0.68 (0.58-0.80) |
| <20 patients | Reference | Reference |
| Immigrants (per 10%) | 1.0 (0.99-1.0) | 1.0 (0.99-1.1) |
| Median Income (per $10k) | 0.82 (0.77-0.88) | 0.83 (0.77-0.89) |
| Percentage aboriginal ancestry |  |  |
| ≥10% | 0.87 (0.77-0.99) | 0.88 (0.78-1.0) |
| 5%-<10% | 0.85 (0.76-0.95) | 0.85 (0.76-0.95) |
| <5% | Reference | Reference |
