# Supplementary material for: Sociodemographic correlates of HIV drug resistance and access to drug resistance testing in British Columbia, Canada
Source: PLoS One. 2017 Sep 22;12(9):e0184848. doi: 10.1371/journal.pone.0184848 (PMC5609746; doi:10.1371/journal.pone.0184848)
Supplement: S2 Table — (DOCX) [file pone.0184848.s007.docx]

| **Multivariable Covariates of Developing Drug Resistance** | **Without Adherence – aHR (95% CI) N=5175** | **With Adherence – aHR (95% CI) N=5175** |
| --- | --- | --- |
| Age |  |  |
| ≥50 Years | 0.72 (0.57-0.91) | 0.82 (0.65-1.0) |
| 40-<50 Years | 0.96 (0.80-1.2) | 1.0 (0.87-1.3) |
| 30-<40 Years | 1.0 (0.88-1.2) | 1.1 (0.95-1.3) |
| <30 Years | Reference | Reference |
| Sex |  |  |
| Female (vs Male) | 1.2 (1.1-1.4) | 1.1 (0.95-1.3) |
| PWID |  |  |
| Yes (vs No) | 1.6 (1.4-1.8) | 1.3 (1.1-1.5) |
| Unknown (vs No) | 1.3 (1.1-1.6) | 1.2 (0.98-1.4) |
| Adherence in first 12 months of therapy <95% (vs ≥95%) | N/A | 2.2 (1.9-2.5) |
| Baseline CD4 |  |  |
| <200 cells/μL | 1.8 (1.5-2.1) | 1.9 (1.6-2.3) |
| 200-<350 cells/μL | 1.3 (1.1-1.5) | 1.3 (1.1-1.6) |
| ≥350 cells/μL | Reference | Reference |
| Baseline pVL |  |  |
| ≥100,000 copies/mL | 2.0 (1.5-2.5) | 2.0 (1.6-2.6) |
| 10,000-<100,000 copies/mL | 1.3 (1.0-1.7) | 1.3 (1.0-1.7) |
| <10,000 copies/mL | Reference | Reference |
| First Year of ARV |  |  |
| 2008-2013 | 0.38 (0.32-0.46) | 0.44 (0.36-0.53) |
| 2004-2007 | 0.48 (0.40-0.56) | 0.52 (0.44-0.62) |
| 2000-2003 | 0.78 (0.67-0.90) | 0.82 (0.71-0.95) |
| 1996-1999 | Reference | Reference |
| Immigrants (per 10%) | Not Selected | 1.1 (1.0-1.1) |
| Median Income (per $10k) | 0.84 (0.77-0.92) | 0.82 (0.76-0.89) |
| Percentage aboriginal ancestry |  |  |
| ≥10% | 1.3 (1.1-1.6) | 1.2 (1.1-1.5) |
| 5%-<10% | 0.89 (0.74-1.1) | 0.87 (0.72-1.1) |
| <5% | Reference | Reference |
