# Supplementary material for: Sociodemographic correlates of HIV drug resistance and access to drug resistance testing in British Columbia, Canada
Source: PLoS One. 2017 Sep 22;12(9):e0184848. doi: 10.1371/journal.pone.0184848 (PMC5609746; doi:10.1371/journal.pone.0184848)
Supplement: S3 Table — Individuals were excluded due to missing data resulting in the inability to link census data to clinical data, therefore we were not able to compare differences between census-level sociodemographic data. Cohorts were compared using Chi-squared test. (DOCX) [file pone.0184848.s008.docx]

| **Multivariable Covariates of Accessing Drug Resistance Testing** | **Included - N(%)**  **N=8398** | **Excluded - N(%) N=1058** | **P-value** |
| --- | --- | --- | --- |
| Sex |  |  | 0.078 |
| Female | 1482 (17.7) | 210 (19.9) |  |
| Male | 6916 (82.4) | 848 (80.2) |  |
| Age at First ARV (years) |  |  | <0.0001 |
| <30 | 1322 (15.74) | 231 (21.83) |  |
| 30-≤39 | 3110 (37.03) | 421 (39.79) |  |
| 40-≤50 | 2619 (31.19) | 279 (26.37) |  |
| >50 | 1347 (16.04) | 127 (12) |  |
| Hepatitis C |  |  | <0.0001 |
| Positive | 3132 (37.29) | 441 (41.68) |  |
| Negative | 4266 (50.8) | 455 (43.01) |  |
| Unknown | 1000 (11.91) | 162 (15.31) |  |
| Baseline regimen third drug class |  |  | <0.0001 |
| NNRTI | 2472 (29.44) | 301 (28.45) |  |
| PI | 3679 (43.81) | 348 (32.89) |  |
| nRTI Only | 1.5 (1.3-1.6) | 1.4 (1.3-1.6) |  |
| Other | 1.2 (0.90-1.6) | 1.2 (0.86-1.5) |  |
| Adherence in first 12 months of therapy |  |  | 0.013 |
| <95% | 3550 (42.27) | 336 (31.76) |  |
| ≥95% | 4543 (54.1) | 353 (33.36) |  |
| Unknown | 305 (3.63) | 369 (34.88) |  |
| Baseline CD4 |  |  | <0.0001 |
| <200 cells/μL | 3273 (38.97) | 288 (27.22) |  |
| 200-349 cells/μL | 2516 (29.96) | 283 (26.75) |  |
| ≥350 cells/μL | 2505 (29.83) | 445 (42.06) |  |
| Unknown | 104 (1.24) | 42 (3.97) |  |
| Baseline pVL |  |  | <0.0001 |
| <9,999 copies/mL | 3273 (38.97) | 288 (27.22) |  |
| 10,000-99,999 copies/mL | 2516 (29.96) | 283 (26.75) |  |
| ≥100,000 copies/mL | 2505 (29.83) | 445 (42.06) |  |
| Unknown | 1532 (18.24) | 367 (34.69) |  |
| Ever DRT (with eligible pVL) |  |  | <0.0001 |
| No | 4271 (50.86) | 463 (43.76) |  |
| Yes | 4127 (49.14) | 595 (56.24) |  |
| Physician experience (last 2 years) |  |  | <0.0001 |
| <20 patients | 2402 (28.6) | 129 (12.19) |  |
| 20-100 patients | 2591 (30.85) | 235 (22.21) |  |
| ≥100 patients | 2685 (31.97) | 285 (26.94) |  |
| Unknown | 720 (8.57) | 409 (38.66) |  |
