# Supplementary material for: Sociodemographic correlates of HIV drug resistance and access to drug resistance testing in British Columbia, Canada
Source: PLoS One. 2017 Sep 22;12(9):e0184848. doi: 10.1371/journal.pone.0184848 (PMC5609746; doi:10.1371/journal.pone.0184848)
Supplement: S4 Table — Individuals with unknown PWID status were excluded from this analysis (N = 1627). (DOCX) [file pone.0184848.s009.docx]

| **Multivariable Covariates of Accessing Drug Resistance Testing** | **Non-PWID – aOR (95% CI)**  **N= 3852** | **PWID aOR – aOR (95% CI)**  **N= 2919** |
| --- | --- | --- |
| Sex |  |  |
| Female (vs Male) | 1.3 (1.1-1.5) | 1.1 (1.0-1.3) |
| Hepatitis C |  |  |
| Positive (vs Negative) | 1.2 (1.1-1.4) | 0.92 (0.80-1.1) |
| Unknown (vs Negative) | 0.38 (0.30-0.47) | 0.35 (0.25-0.49) |
| Baseline regimen third drug class |  |  |
| PI (vs NNRTI) | 1.2 (1.1-1.4) | 0.95 (0.84-1.1) |
| nRTI Only (vs NNRTI) | 2.1 (1.7-2.6) | 1.2 (0.97-1.4) |
| Other (vs NNRTI) | 1.6 (1.1-2.3) | 1.2 (0.65-2.3) |
| Adherence in first 12 months of therapy <95% (vs ≥95%) | 1.3 (1.2-1.5) | 1.2 (1.1-1.3) |
| Baseline CD4 |  |  |
| <200 cells/μL | 1.6 (1.4-1.8) | 1.3 (1.2-1.5) |
| 200-<350 cells/μL | 1.2 (1.0-1.3) | 1.1 (0.99-1.3) |
| ≥350 cells/μL | Reference | Reference |
| Baseline pVL |  |  |
| ≥100,000 copies/mL | 1.3 (1.1-1.6) | 1.2 (1.0-1.5) |
| 10,000-<100,000 copies/mL | 0.97 (0.80-1.2) | 1.1 (0.90-1.3) |
| <10,000 copies/mL | Reference | Reference |
| Eligible for drug resistance test (per year) | 1.1 (1.1-1.1) | 1.1 (1.1-1.1) |
| Physician experience (last 2 years) |  |  |
| ≥100 patients | 0.92 (0.80-1.1) | Not Selected |
| 20-100 patients | 1.1 (0.93-1.2) | Not Selected |
| Unknown | 0.71 (0.53-0.95) | Not Selected |
| <20 patients | Reference | Not Selected |
| Immigrants (per 10%) | Not Selected | 1.1 (1.0-1.1) |
| Median Income (per $10k) | 0.84 (0.77-0.92) | 0.82 (0.76-0.89) |
| Percentage aboriginal ancestry |  |  |
| ≥10% | 0.73 (0.58-0.91) | Not Selected |
| 5%-<10% | 0.88 (0.75-1.0) | Not Selected |
| <5% | Reference | Not Selected |
