# Supplementary material for: Sociodemographic correlates of HIV drug resistance and access to drug resistance testing in British Columbia, Canada
Source: PLoS One. 2017 Sep 22;12(9):e0184848. doi: 10.1371/journal.pone.0184848 (PMC5609746; doi:10.1371/journal.pone.0184848)
Supplement: S5 Table — Individuals with unknown PWID status were excluded from this analysis (N = 962). (DOCX) [file pone.0184848.s010.docx]

| **Multivariable Covariates of Developing Drug Resistance** | **Non-PWID - aHR (95% CI) N=2325** | **PWID – aHR (95% CI) N=1888** |
| --- | --- | --- |
| Age |  |  |
| >50 Years | 0.87 (0.59-1.3) | 0.64 (0.45-0.91) |
| 40-<50 Years | 1.2 (0.82-1.6) | 0.91 (0.71-1.2) |
| 30-<40 Years | 1.4 (1.0-2.0) | 0.96 (0.77-1.2) |
| <30 Years | Reference | Reference |
| Sex |  |  |
| Female (vs Male) | 1.0 (0.76-1.4) | Not Selected |
| Hepatitis C |  |  |
| Positive (vs Negative) | Not Selected | 1.1 (0.87-1.5) |
| Unknown (vs Negative) | Not Selected | 2.0 (1.1-3.7) |
| Baseline regimen third drug class |  |  |
| PI (vs NNRTI) | 1.2 (0.98-1.5) | 0.76 (0.64-0.91) |
| Adherence in first 12 months of therapy <95% (vs ≥95%) | 2.6 (2.1-3.1) | 1.9 (1.6-2.3) |
| Baseline CD4 |  |  |
| <200 cells/μL | 1.7 (1.3-2.2) | 2.2 (1.7-2.7) |
| 200-<350 cells/μL | 1.3 (0.98-1.8) | 1.3 (1.0-1.7) |
| ≥350 cells/μL | Reference | Reference |
| Baseline pVL |  |  |
| ≥100,000 copies/mL | 1.6 (1.1-2.5) | 2.5 (1.8-3.5) |
| 10,000-<100,000 copies/mL | 1.0 (0.67-1.6) | 1.7 (1.2-2.5) |
| <10,000 copies/mL | Reference | Reference |
| First Year on cART |  |  |
| 2008-2013 | 0.41 (0.30-0.56) | 0.64 (0.49-0.83) |
| 2004-2007 | 0.63 (0.47-0.84) | 0.49 (0.39-0.63) |
| 2000-2003 | 0.88 (0.68-1.1) | 0.74 (0.59-0.92) |
| 1996-1999 | Reference | Reference |
| Immigrants (per 10%) | 1.1 (0.99-1.1) | Not Selected |
| Percentage aboriginal ancestry |  |  |
| ≥10% | Not Selected | 1.3 (1.1-1.5) |
| 5%-<10% | Not Selected | 0.90 (0.70-1.2) |
| <5% | Not Selected | Reference |
