# Supplementary material for: Sociodemographic correlates of HIV drug resistance and access to drug resistance testing in British Columbia, Canada
Source: PLoS One. 2017 Sep 22;12(9):e0184848. doi: 10.1371/journal.pone.0184848 (PMC5609746; doi:10.1371/journal.pone.0184848)
Supplement: S6 Table — Individuals were excluded due to missing data resulting in the inability to link census-level sociodemographic data to individual clinical data, therefore we were not able to compare differences between census-level sociodemographic data. Cohorts were compared using Chi-squared test. (DOCX) [file pone.0184848.s011.docx]

| **Multivariable Covariates of Developing Drug Resistance** | **Included - N(%) N=5175** | **Excluded - N(%) N=528** | **P-value** |
| --- | --- | --- | --- |
| Sex |  |  | <0.0001 |
| Female | 943 (18.22) | 135 (25.57) |  |
| Male | 4232 (81.78) | 393 (74.43) |  |
| Age at First ARV (years) |  |  | 0.0006 |
| <30 | 669 (12.93) | 100 (18.94) |  |
| 30-<40 | 1799 (34.76) | 183 (34.66) |  |
| 40-<50 | 1743 (33.68) | 167 (31.63) |  |
| >50 | 964 (18.63) | 78 (14.77) |  |
| Hepatitis C |  |  | <0.0001 |
| Positive | 2109 (40.75) | 285 (53.98) |  |
| Negative | 2734 (52.83) | 203 (38.45) |  |
| Unknown | 332 (6.42) | 40 (7.58) |  |
| Baseline regimen third drug class |  |  | 0.0009 |
| NNRTI | 2111 (40.79) | 255 (48.3) |  |
| PI | 3064 (59.21) | 273 (51.7) |  |
| Adherence in first 12 months of therapy |  |  | 0.0001 |
| <95% | 1960 (37.87) | 245 (46.4) |  |
| ≥95% | 3215 (62.13) | 283 (53.6) |  |
| Baseline CD4 |  |  | 0.0002 |
| <200 cells/μL | 2310 (44.64) | 212 (40.15) |  |
| 200-<350 cells/μL | 1558 (30.11) | 139 (26.33) |  |
| ≥350 cells/μL | 1307 (25.26) | 177 (33.52) |  |
| Baseline pVL |  |  | 0.0022 |
| <10,000 copies/mL | 553 (10.69) | 78 (14.77) |  |
| 10,000-<100,000 copies/mL | 2118 (40.93) | 229 (43.37) |  |
| ≥100,000 copies/mL | 2504 (48.39) | 221 (41.86) |  |
| Ever DRT (with eligible pVL) |  |  | 0.018 |
| No | 2904 (56.12) | 268 (50.76) |  |
| Yes | 2271 (43.88) | 260 (49.24) |  |
| Physician experience (last 2 years) |  |  | <0.0001 |
| <20 patients | 1402 (27.09) | 96 (18.18) |  |
| 20-100 patients | 1637 (31.63) | 177 (33.52) |  |
| ≥100 patients | 1944 (37.57) | 239 (45.27) |  |
| Unknown | 192 (3.71) | 16 (3.03) |  |
