# Supplementary material for: Sociodemographic correlates of HIV drug resistance and access to drug resistance testing in British Columbia, Canada
Source: PLoS One. 2017 Sep 22;12(9):e0184848. doi: 10.1371/journal.pone.0184848 (PMC5609746; doi:10.1371/journal.pone.0184848)
Supplement: S7 Table — (DOCX) [file pone.0184848.s012.docx]

| **Multivariable Covariates of Accessing Drug Resistance Testing** | **Univariable**  **OR (95% CI) N=8398** | **Multivariable**  **aOR (95% CI) N=8398** |
| --- | --- | --- |
| Age |  |  |
| ≥50 Years of age | 1.9 (1.7-2.2) | Not Selected |
| 40-<50 Years of age | 1.5 (1.3-1.7) | Not Selected |
| 30-<40 Years of age | 1.2 (1.1-1.4) | Not Selected |
| <30 Years of age | Reference | Not Selected |
| Sex |  |  |
| Female (vs Male) | 1.3 (1.2-1.4) | 1.2 (1.1-1.3) |
| MSM Risk |  |  |
| MSM (vs non-MSM) | 1.0 (0.94-1.1) | Not Selected |
| MSM risk unknown (vs non-MSM) | 0.43 (0.39-0.48) | Not Selected |
| Heterosexual Risk |  |  |
| Heterosexual (vs non-heterosexual) | 1.4 (1.3-1.5) | Not Selected |
| Heterosexual risk unknown | 0.54 (0.49-0.59) | Not Selected |
| PWID Risk |  |  |
| PWID (vs non-PWID) | 1.2 (1.1-1.3) | 1.1 (1.0-1.2) |
| PWID risk unknown (vs non-PWID) | 0.43 (0.38-0.48) | 0.47 (0.42-0.53) |
| Hepatitis C |  |  |
| Positive (vs Negative) | 1.2 (1.1-1.3) | Not Selected |
| Unknown (vs Negative) | 0.44 (0.38-0.51) | Not Selected |
| Baseline regimen third drug class |  |  |
| PI (vs NNRTI) | 1.0 (0.94-1.1) | 1.1 (0.97-1.1) |
| nRTI Only (vs NNRTI) | 1.3 (1.2-1.4) | 1.4 (1.3-1.6) |
| Other (vs NNRTI) | 1.3 (0.97-1.7) | 1.2 (0.86-1.5) |
| Adherence |  |  |
| First 12 months of therapy <95% | 1.3 (1.2-1.4) | 1.3 (1.2-1.4) |
| First 12 months of therapy Unknown | 1.7 (1.5-2.1) | 2.6 (2.1-3.3) |
| First 12 months of therapy >95% | Reference | Reference |
| Baseline CD4 |  |  |
| <200 cells/μL | 1.4 (1.3-1.5) | 1.5 (1.3-1.6) |
| 200-<350 cells/μL | 1.2 (1.1-1.3) | 1.2 (1.1-1.3) |
| Unknown | 1.3 (1.0-1.6) | 0.94 (0.72-1.2) |
| ≥350 cells/μL | Reference | Reference |
| Baseline pVL |  |  |
| ≥100,000 copies/mL | 1.1 (0.98-1.2) | 1.2 (1.1-1.4) |
| 10,000-<100,000 copies/mL | 0.94 (0.84-1.1) | 1.0 (0.90-1.2) |
| Unknown (First ARV before 1997) | 1.5 (1.3-1.7) | 1.8 (1.5-2.1) |
| Unknown (Other) | 2.0 (1.5-2.6) | 2.2 (1.6-2.9) |
| <10,000 copies/mL | Reference | Reference |
| Year eligible for drug resistance test (per year) | 1.1 (1.1-1.1) | 1.1 (1.1-1.1) |
| Physician experience (last 2 years) |  |  |
| ≥100 patients | 0.75 (0.69-0.82) | 0.92 (0.84-1.0) |
| 20-100 patients | 1.1 (0.97-1.1) | 1.1 (0.98-1.2) |
| Unknown | 0.88 (0.78-1.0) | 0.68 (0.58-0.80) |
| <20 patients | Reference | Reference |
| One-family households (per 10%) | 1.0 (0.99-1.0) | Not Selected |
| Population density (per 10k) | 1.0 (0.95-1.1) | Not Selected |
| Immigrants (per 10%) | 1.0 (1.0-1.0) | 1.0 (0.99-1.1) |
| Median Income (per $10k) | 0.87 (0.83-0.91) | 0.83 (0.77-0.89) |
| Single people (per 10%) | 1.0 (1.0-1.0) | Not Selected |
| Post-secondary certificate (per 10%) | 0.94 (0.92-0.97) | Not Selected |
| Unemployment rate (per 10%) | 0.97 (0.94-1.0) | Not Selected |
| Percentage aboriginal ancestry |  |  |
| ≥10% | 1.1 (1.0-1.2) | 0.88 (0.78-1.0) |
| 5%-<10% | 1.0 (0.92-1.1) | 0.85 (0.76-0.95) |
| <5% | Reference | Reference |
