# Supplementary material for: Sociodemographic correlates of HIV drug resistance and access to drug resistance testing in British Columbia, Canada
Source: PLoS One. 2017 Sep 22;12(9):e0184848. doi: 10.1371/journal.pone.0184848 (PMC5609746; doi:10.1371/journal.pone.0184848)
Supplement: S8 Table — (DOCX) [file pone.0184848.s013.docx]

| **Multivariable Covariates of Developing Drug Resistance** | **Univariable**  **HR (95% CI) N=5175** | **Multivariable**  **aHR (95% CI) N=5175** |
| --- | --- | --- |
| Age |  |  |
| ≥50 Years of age | 0.60 (0.48-0.76) | 0.82 (0.65-1.0) |
| 40-<50 Years of age | 0.90 (0.75-1.1) | 1.0 (0.87-1.3) |
| 30-<40 Years of age | 1.1 (0.90-1.3) | 1.1 (0.95-1.3) |
| <30 Years of age | Reference | Reference |
| Sex |  |  |
| Female (vs Male) | 1.3 (1.2-1.5) | 1.1 (0.95-1.3) |
| MSM Risk |  |  |
| MSM (vs non-MSM) | 0.76 (0.67-0.87) | Not Selected |
| MSM risk unknown (vs non-MSM) | 0.53 (0.45-0.62) | Not Selected |
| Heterosexual Risk |  |  |
| Heterosexual (vs non-heterosexual) | 1.1 (0.95-1.2) | Not Selected |
| Heterosexual risk unknown | 0.66 (0.57-0.77) | Not Selected |
| PWID Risk |  |  |
| PWID (vs non-PWID) | 1.8 (1.6-2.0) | 1.3 (1.1-1.5) |
| PWID risk unknown (vs non-PWID) | 1.2 (1.0-1.5) | 1.2 (0.98-1.4) |
| Hepatitis C |  |  |
| Positive (vs Negative) | 1.8 (1.6-2.0) | Not Selected |
| Unknown (vs Negative) | 1.2 (0.89-1.6) | Not Selected |
| Baseline regimen third drug class |  |  |
| PI (vs NNRTI) | 1.2 (1.0-1.3) | Not Selected |
| Adherence |  |  |
| First 12 months of therapy <95% (vs >95%) | 2.6 (2.3-2.9) | 2.2 (1.9-2.5) |
| Baseline CD4 |  |  |
| <200 cells/μL | 1.9 (1.6-2.2) | 1.9 (1.6-2.3) |
| 200-<350 cells/μL | 1.1 (0.93-1.3) | 1.3 (1.1-1.6) |
| ≥350 cells/μL | Reference | Reference |
| Baseline pVL |  |  |
| ≥100,000 copies/mL | 2.6 (2.1-3.3) | 2.0 (1.6-2.6) |
| 10,000-<100,000 copies/mL | 1.4 (1.1-1.8) | 1.3 (1.0-1.7) |
| <10,000 copies/mL | Reference | Reference |
| First year of ARV |  |  |
| 2008-2013 | 0.31 (0.25-0.37) | 0.44 (0.36-0.53) |
| 2004-2007 | 0.53 (0.45-0.63) | 0.52 (0.44-0.62) |
| 2000-2003 | 0.89 (0.77-1.0) | 0.82 (0.71-0.95) |
| 1996-1999 | Reference | Reference |
| Physician experience (last 2 years) |  |  |
| ≥100 patients | 0.74 (0.64-0.86) | Not Selected |
| 20-100 patients | 0.98 (0.85-1.1) | Not Selected |
| Unknown | 0.98 (0.70-1.4) | Not Selected |
| <20 patients | Reference | Not Selected |
| One-family households (per 10%) | 0.97 (0.95-1.0) | Not Selected |
| Population density (per 10k) | 0.89 (0.82-0.97) | Not Selected |
| Immigrants (per 10%) | 0.97 (0.94-1.0) | Not Selected |
| Median Income (per $10k) | 0.65 (0.60-0.71) | Not Selected |
| Single people (per 10%) | 1.0 (1.0-1.1) | Not Selected |
| Post-secondary certificate (per 10%) | 0.81 (0.77-0.85) | Not Selected |
| Unemployment rate (per 10%) | 0.82 (0.78-0.86) | Not Selected |
| Percentage aboriginal ancestry |  |  |
| ≥10% | 1.5 (1.3-1.8) | 1.2 (1.1-1.5) |
| 5%-<10% | 0.85 (0.71-1.0) | 0.87 (0.72-1.1) |
| <5% | Reference | Reference |
